# Reassessing the Role of Anthracyclines in Early HER2-Positive Breast Cancer in the Era of HER2-Targeted Therapy and Treatment De-Escalation: A Systematic Review and Narrative Synthesis

**DOI:** 10.3390/jcm15187303

**Published:** 2026-09-20

**Authors:** Zhuldyz Myrzabay, Nazym Askambayeva, Ahmad J. Harahsheh, Reezal Ishak, Mohamad Aljofan

**Affiliations:** 1 Department of Biomedical Sciences, School of Medicine, Nazarbayev University, 5/1 Kerey and Zhanibek Khandar Str., 010000 Astana, Kazakhstan; zhuldyz.myrzabay@nu.edu.kz (Z.M.); nazym.askambayeva@nu.edu.kz (N.A.); 2 Department of Biology and Biotechnology, Faculty of Science, The Hashemite University, Zarqa 13133, Jordan; ahmad_harahsheh@hu.edu.jo; 3 Institute of Medical Science Technology (UniKL MESTECH), Universiti Kuala Lumpur, A1-1, Jalan TKS 1, Taman Kajang Sentral, Kajang 43000, Selangor, Malaysia; reezal@unikl.edu.my; 4 National Laboratory Astana, 010000 Astana, Kazakhstan

**Keywords:** HER2-positive breast cancer, trastuzumab, cardiotoxicity, treatment de-escalation, anthracycline-free regimens

## Abstract

**Background:** Anthracyclines have historically remained a cornerstone of treatment for early HER2-positive breast cancer. However, the emergence of dual HER2 blockade, antibody–drug conjugates, and response-adapted treatment strategies has changed the clinical context in which anthracycline use is considered. Whether anthracyclines continue to provide sufficient oncologic benefit to justify their additional cardiovascular toxicity remains uncertain. **Objectives:** This study aims to evaluate the contemporary role of anthracyclines in early HER2-positive breast cancer by synthesizing direct comparative evidence and contextual evidence from prospective de-escalation studies. **Design:** It involves a systematic review with narrative synthesis. **Data Sources and Methods**: PubMed, the Web of Science, and CENTRAL were searched for prospective studies published from January 2000 to 31 May 2026. A meta-analysis was initially planned, but only three clinically heterogeneous trials directly compared anthracycline-containing and anthracycline-free regimens. Direct comparative and contextual de-escalation evidence was therefore synthesized separately using a narrative approach. **Results:** Twelve studies were included. BCIRG-006, TRAIN-2, and TRYPHAENA provided direct comparative evidence; nine additional prospective trials provided contextual evidence on anthracycline-sparing or treatment de-escalation strategies. Direct comparisons generally indicated greater cardiac toxicity with anthracycline-containing regimens, although severe events were uncommon and cardiotoxicity definitions varied. Contextual studies supported the feasibility of contemporary anthracycline-sparing approaches in selected populations but did not provide direct estimates of the effect of anthracycline exposure. **Conclusions:** Anthracycline-containing regimens appear to confer greater cardiac risk than anthracycline-free regimens, but comparative evidence remains limited. The available evidence supports selective rather than routine anthracycline use, with treatment decisions guided by the anticipated oncologic benefit, cardiovascular risk, tumor characteristics, treatment setting, and patient preferences. **Registration:** PROSPERO CRD420261296599; registered on 29 January 2026.

## 1. Introduction

The optimal role of anthracyclines in the treatment of early human epidermal growth factor receptor 2 (HER2)-positive breast cancer has become increasingly uncertain in the era of modern HER2-directed therapy. Although anthracyclines remain an important component of systemic treatment, the necessity of their routine use in all patients has been questioned as less cardiotoxic HER2-directed treatment strategies have demonstrated favorable clinical outcomes in selected patient populations. Breast cancer remains the most frequently diagnosed cancer among women worldwide, and HER2-positive disease accounts for approximately 15–20% of all breast cancers [1,2]. Although anthracyclines historically formed the backbone of systemic therapy, advances in HER2-targeted treatment have substantially transformed the therapeutic landscape.

The introduction of trastuzumab fundamentally changed the prognosis of HER2-positive breast cancer. By targeting the extracellular domain of the HER2 receptor, trastuzumab significantly improved disease-free and overall survival and became a cornerstone of treatment in both early-stage and advanced disease [2,3,4]. More recently, the development of dual HER2 blockade, antibody–drug conjugates, and treatment de-escalation strategies has further expanded therapeutic options and challenged the routine use of anthracycline-containing regimens.

Despite these advances, cardiotoxicity remains one of the most clinically relevant adverse effects of HER2-directed therapy. Cardiac dysfunction may present as an asymptomatic decline in left ventricular ejection fraction (LVEF) or, less commonly, symptomatic heart failure. As survival outcomes continue to improve, preservation of long-term cardiovascular health has become an increasingly important component of treatment decision-making and survivorship care.

Anthracyclines represent an additional source of cardiovascular risk. Unlike trastuzumab-associated cardiac dysfunction, anthracycline-induced cardiotoxicity is cumulative, dose-dependent, and may result in irreversible myocardial injury. Proposed mechanisms include oxidative stress, mitochondrial dysfunction, and cardiomyocyte death. Prior exposure to anthracyclines may therefore increase susceptibility to cardiac dysfunction during subsequent HER2-targeted therapy. These concerns have contributed to growing interest in anthracycline-sparing treatment strategies that aim to reduce toxicity while maintaining oncologic efficacy [5].

Although trastuzumab-based regimens are widely used in the management of early HER2-positive breast cancer, the magnitude of cardiotoxicity directly attributable to anthracycline exposure remains incompletely defined. Interpretation of existing evidence is complicated by differences in treatment sequencing, cardiotoxicity definitions, and cardiac monitoring approaches across clinical trials. Consequently, uncertainty remains regarding which patients derive sufficient oncologic benefit from anthracyclines to justify their additional cardiovascular risk within the contemporary HER2-directed treatment landscape.

A previous systematic review and meta-analysis by Zhu et al. evaluated anthracycline-containing versus anthracycline-free neoadjuvant regimens in HER2-positive breast cancer and reported comparable pathological complete responses with lower cardiotoxicity for anthracycline-free approaches [6]. However, that review primarily addressed comparative neoadjuvant treatment regimens and focused on pathological responses and treatment-related toxicity. Since then, the therapeutic landscape of early HER2-positive breast cancer has continued to evolve with the incorporation of dual HER2 blockade, response-guided treatment strategies, antibody–drug conjugates, and increasing interest in treatment de-escalation. Furthermore, several pivotal prospective trials have expanded the evidence base supporting treatment de-escalation, response-guided therapy, and individualized therapeutic strategies, extending beyond the scope of earlier comparative analyses. Importantly, that review was designed to compare neoadjuvant treatment regimens rather than to reassess the broader contemporary role of anthracyclines across the evolving spectrum of HER2-directed treatment strategies. These developments have shifted the contemporary clinical question beyond whether anthracyclines improve pathological response toward identifying which patients continue to derive sufficient oncologic benefit to justify their additional cardiovascular risk.

Therefore, this systematic review aimed to reassess the contemporary role of anthracyclines in early HER2-positive breast cancer by integrating direct comparative evidence with the broader clinical context of modern HER2-directed treatment strategies. We synthesized evidence from studies directly comparing anthracycline-containing and anthracycline-free regimens while also incorporating prospective evidence from contemporary anthracycline-sparing, response-adapted, and treatment de-escalation trials to provide a comprehensive assessment of the current role of anthracyclines. Owing to the limited number of direct comparative studies and considerable clinical and methodological heterogeneity, the evidence was synthesized narratively rather than through quantitative meta-analysis.

## 2. Methods

### 2.1. Study Design

This systematic review evaluated the role of anthracyclines in patients with early HER2-positive breast cancer receiving HER2-targeted therapy. A quantitative meta-analysis was initially planned. However, after full-text assessment and data extraction, only three trials were found to provide direct comparisons of anthracycline-containing and anthracycline-free regimens. Substantial heterogeneity was also identified in treatment setting, chemotherapy backbone, HER2-targeted therapy, definitions of cardiotoxicity, cardiac monitoring, and follow-up. Therefore, a structured narrative synthesis was performed. Direct comparative trials formed the primary evidence base, while prospective trials of contemporary anthracycline-sparing and treatment de-escalation strategies were synthesized separately as contextual evidence. The review was conducted and reported according to the Preferred Reporting Items for Systematic Reviews and Meta-Analyses (PRISMA 2020) statement [7]. The review protocol was prospectively registered in PROSPERO on 29 January 2026 (CRD420261296599). The change from quantitative to narrative synthesis is reported transparently as a deviation from the original analytical plan.

### 2.2. Search Strategy

We performed a comprehensive search in three electronic databases: PubMed, Web of Science, and CENTRAL. We conducted the initial database searches on 20 February 2026 and updated all three databases on 31 May 2026 to identify additional eligible studies published or indexed since the initial search. The search covered publications from January 2000 to 31 May 2026, corresponding to the contemporary era of trastuzumab-based treatment and anthracycline-containing chemotherapy. We sought to identify studies evaluating cardiotoxicity outcomes in patients with early HER2-positive breast cancer treated with trastuzumab-containing regimens, with or without anthracyclines. The search strategy combined terms related to HER2-positive breast cancer, trastuzumab, anthracyclines, cardiotoxicity, heart failure, and LVEF. Only English-language studies were included. We limited the search to PubMed, Web of Science, and CENTRAL, supplemented by manual screening of the reference lists of relevant articles; we did not systematically search Embase or trial registries. We did not search conference proceedings separately because conference abstracts were excluded under the eligibility criteria, which required sufficient published study-level data to assess cardiac outcomes. We applied search terms as free-text keywords across the title, abstract, topic, and keyword fields, as appropriate for each database. Controlled-vocabulary terms, including PubMed Medical Subject Headings (MeSH), were not additionally applied. The complete database-specific search strategies are provided in Supplementary Table S1.

### 2.3. Eligibility Criteria

We defined the eligibility criteria according to the PICOS framework. The population of interest included adult patients with early or locally advanced HER2-positive breast cancer receiving neoadjuvant or adjuvant HER2-targeted therapy.

We classified eligible studies into two evidence groups. The primary evidence group comprised randomized or prospective trials directly comparing anthracycline-containing and anthracycline-free regimens. The contextual evidence group comprised prospective trials evaluating contemporary anthracycline-sparing, response-adapted, dual HER2-blockade, antibody–drug conjugate, or other treatment de-escalation strategies. Contextual studies were eligible if they enrolled patients with early or locally advanced HER2-positive breast cancer and reported relevant cardiac safety outcomes, even when they did not include a direct anthracycline comparator.

The primary outcomes of interest included LVEF decline, symptomatic heart failure, symptomatic left ventricular systolic dysfunction, and other treatment-related cardiac adverse events. Secondary outcomes included pathological complete response, invasive disease-free survival, event-free survival, overall survival, and findings relevant to treatment de-escalation and contemporary HER2-targeted therapy.

Randomized controlled trials and prospective clinical studies published in English between January 2000 and 31 May 2026 were eligible. Studies in the primary evidence group were required to provide a direct comparison between anthracycline-containing and anthracycline-free treatment strategies. Studies in the contextual evidence group were required to evaluate a contemporary anthracycline-sparing or treatment de-escalation strategy and to report sufficient cardiac outcome data for qualitative synthesis. The two evidence groups were synthesized separately and were not treated as equivalent comparative evidence. Studies involving metastatic breast cancer, retrospective observational studies, review articles, editorials, conference abstracts, case reports, animal studies, and studies lacking relevant cardiac outcome data were excluded.

### 2.4. Study Selection Procedure

A quantitative meta-analysis was initially planned. However, only BCIRG-006, TRAIN-2, and TRYPHAENA directly compared anthracycline-containing and anthracycline-free regimens. These trials differed in clinical setting, treatment backbone, HER2-targeted therapy, definitions of cardiotoxicity, cardiac monitoring, and duration of follow-up. Pooling this limited and heterogeneous evidence was therefore considered unlikely to produce a clinically interpretable summary estimate. A structured narrative synthesis was performed instead. Conclusions regarding the comparative effects of anthracycline exposure were based on the three direct comparative trials. The remaining prospective trials were synthesized separately to describe the cardiac safety and clinical feasibility of contemporary anthracycline-sparing and treatment de-escalation strategies and were not interpreted as direct comparative evidence. An effect direction plot was developed as a qualitative graphical summary; no pooled effect estimate was calculated.

### 2.5. Data Extraction

Data were extracted from the included studies using a standardized data extraction approach. The following information was collected: first author, year of publication, study design, country or setting, patient population, sample size, treatment groups, duration of follow-up, and cardiac outcomes assessed. Two reviewers (Z.M. and N.A.) extracted data independently, as described in the Reliability Section. Extracted data were cross-checked against the original study reports prior to inclusion in the analysis to ensure accuracy.

### 2.6. Outcomes

The primary outcomes of interest included cardiac safety endpoints reported in the eligible studies, including LVEF decline, symptomatic heart failure, symptomatic left ventricular systolic dysfunction, and other treatment-related cardiovascular adverse events. Secondary outcomes included pathological complete response, invasive disease-free survival, event-free survival, overall survival, and findings related to treatment de-escalation and anthracycline-sparing strategies.

### 2.7. Risk-of-Bias Assessment Methods

Risk of bias was assessed independently by two reviewers (Z.M. and N.A.) using design-specific tools. Randomized controlled trials were evaluated using the Cochrane Risk of Bias 2 (RoB 2) tool. Prospective non-randomized intervention studies were evaluated using the Joanna Briggs Institute Critical Appraisal Checklist for Quasi-Experimental Studies. Disagreements between reviewers were resolved through discussion and, when necessary, consultation with a senior investigator. Detailed assessments are presented in Supplementary Tables S2 and S3.

### 2.8. Reliability

Study selection, data extraction, and risk-of-bias assessment were performed independently by two reviewers (Z.M. and N.A.). Reviewers resolved disagreements through discussion and, when necessary, consultation with a senior investigator (M.A.).

### 2.9. Synthesis Approach

The narrative synthesis was conducted in two stages. First, BCIRG-006, TRAIN-2, and TRYPHAENA were synthesized as direct comparative evidence on the cardiac safety and oncologic efficacy of anthracycline-containing versus anthracycline-free regimens. Second, the remaining prospective trials were synthesized as contextual evidence on contemporary anthracycline-sparing and treatment de-escalation strategies. Comparative treatment-effect statements were restricted to trials with an appropriate anthracycline-free comparator. Differences in treatment setting, patient population, chemotherapy backbone, HER2-targeted therapy, definitions of cardiotoxicity, cardiac monitoring, and follow-up were examined narratively. An effect direction plot provided a qualitative summary of study-specific cardiac safety findings without statistical pooling. This narrative synthesis followed the Synthesis Without Meta-analysis (SWiM) reporting guideline [8], which complements PRISMA 2020 [7] for the reporting of synthesis without meta-analysis. We grouped studies into direct comparative and contextual evidence (Section 2.3 and Section 2.4) and used cardiac outcomes, including LVEF decline and clinically overt heart failure or left ventricular systolic dysfunction (LVSD), to structure the synthesis. Section 2.1 explains the rationale for a narrative rather than quantitative synthesis. Clinical and methodological heterogeneity in treatment setting, treatment regimen, cardiotoxicity definitions, cardiac monitoring, and follow-up was examined narratively. We considered risk-of-bias assessments when interpreting the findings. Results are presented in Table 1 and Table 2 and summarized graphically using an effect direction plot. Limitations of the narrative synthesis are discussed in Section 4.1.

### 2.10. Certainty-of-Evidence Assessment

We assessed the certainty of evidence for the key cardiac outcomes in the direct comparative evidence base using the GRADE (Grading of Recommendations Assessment, Development and Evaluation) framework. Certainty was evaluated across five domains, namely risk of bias, inconsistency, indirectness, imprecision, and publication bias, and was rated as high, moderate, low, or very low.

## 3. Results

### 3.1. Study Selection

The literature search identified a total of 189 records from three electronic databases: PubMed (*n* = 52), Web of Science (*n* = 70), and CENTRAL (*n* = 67). After removing 73 duplicate records, 116 studies remained for title and abstract screening. During the screening process, 82 studies were excluded as they were not relevant to the research question, leaving 34 reports for further assessment. Of these, 2 reports could not be retrieved, and 32 full-text articles were assessed for eligibility.

**Figure 1:**
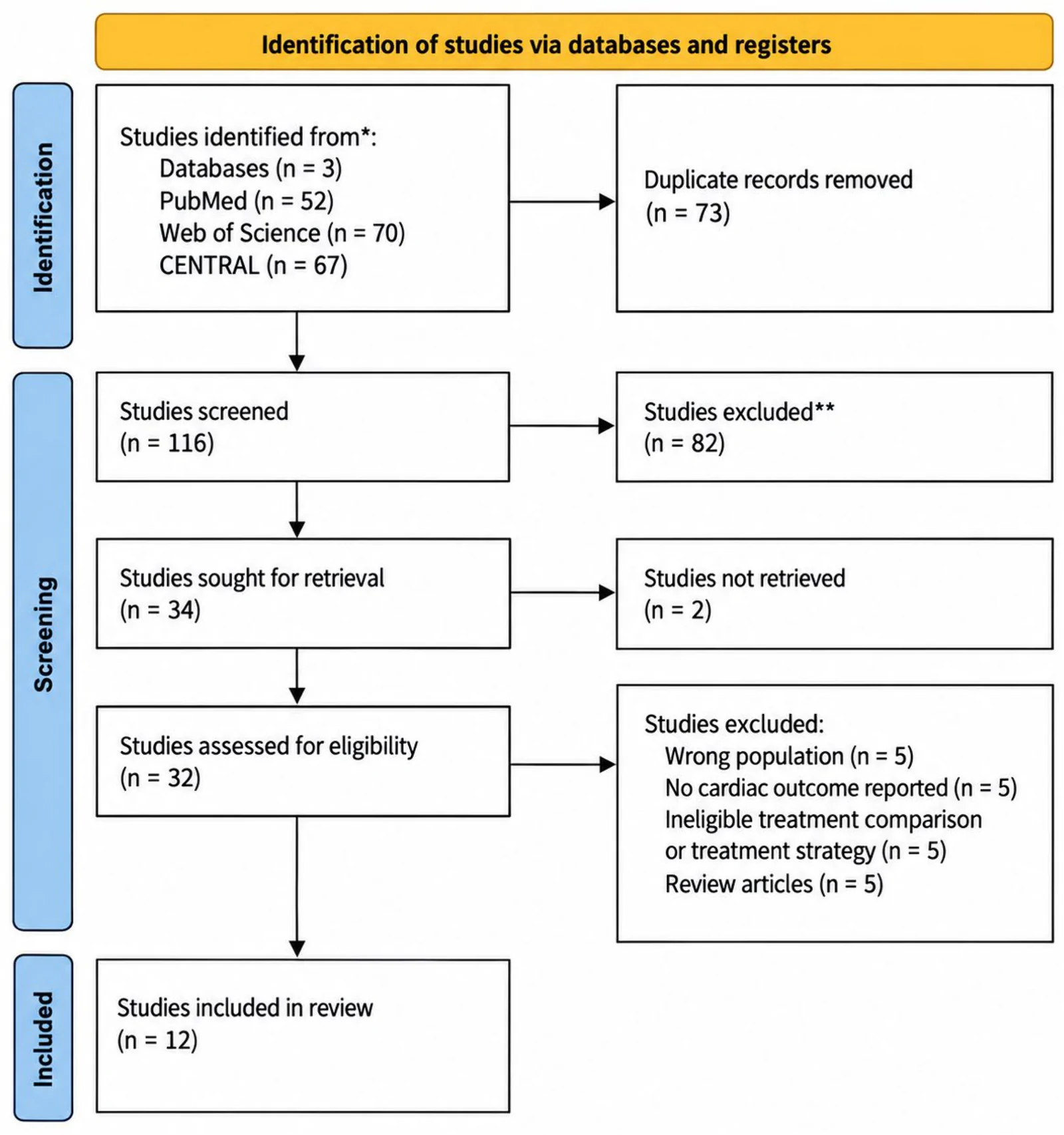
PRISMA flow diagram of study selection. * Studies were identified from three databases: PubMed, Web of Science, and CENTRAL. ** Records were excluded during title and abstract screening.

Among the 32 full-text reports assessed for eligibility, 20 were excluded for the following reasons: ineligible population (*n* = 5), absence of relevant cardiac outcomes (*n* = 5), ineligible treatment comparison or treatment strategy (*n* = 5), and ineligible publication type, including review articles and conference abstracts (*n* = 5). Ultimately, 12 studies met the inclusion criteria and were included in the systematic review with narrative synthesis. The study selection process is illustrated in Figure 1.

The diagram shows the identification, screening, eligibility assessment, and inclusion of studies in the systematic review.

### 3.2. Study Characteristics

A total of 12 studies were included in this systematic review. Three trials, namely BCIRG-006, TRAIN-2, and TRYPHAENA, directly compared anthracycline-containing and anthracycline-free regimens and constituted the primary comparative evidence base. The remaining nine prospective trials evaluated contemporary HER2-targeted, anthracycline-sparing, response-adapted, or treatment de-escalation strategies and were treated as contextual evidence. These studies spanned neoadjuvant and adjuvant settings and were not used to estimate the direct comparative effect of anthracycline exposure. The characteristics of all included studies are summarized in Table 1. Definitions of cardiotoxicity, cardiac assessment methods, monitoring schedules, and follow-up duration for the direct comparative trials are presented in Table 2.

The included studies evaluated a broad spectrum of contemporary HER2-targeted treatment strategies, including conventional anthracycline-containing regimens, anthracycline-free chemotherapy, dual HER2 blockade, response-adapted treatment de-escalation, and antibody–drug conjugates. Cardiac outcomes included symptomatic heart failure, left ventricular ejection fraction decline, and other treatment-related cardiovascular adverse events.

### 3.3. Risk-of-Bias Assessment

**Figure 2:**
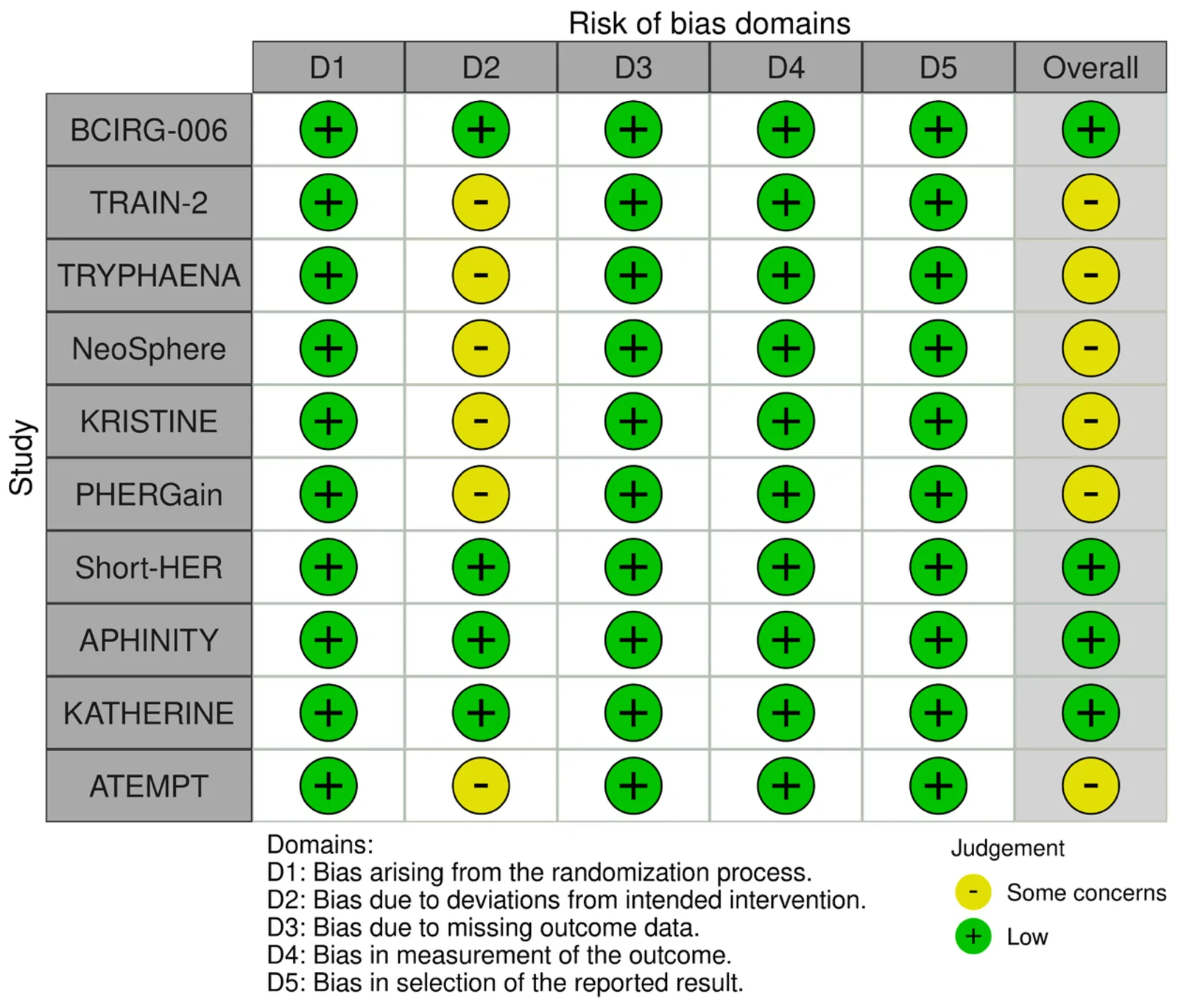
Traffic-light plot of risk-of-bias assessment using the Cochrane Risk of Bias 2 (RoB 2) tool.

Among the 10 randomized controlled trials, the overall risk of bias was judged as low in four trials and as presenting some concerns in six trials. The most frequent concerns were identified in the domain addressing deviations from intended interventions, particularly in open-label trials in which knowledge of treatment allocation could potentially have influenced treatment delivery or subsequent clinical management. Risk of bias in outcome measurement was generally considered low because the principal cardiac outcomes were based on objective assessments, including LVEF measurements and clinically documented heart failure. The domain-level RoB 2 assessments are summarized in Figure 2, with detailed judgments provided in Supplementary Table S2. The two prospective non-randomized studies, BERENICE and APT, were appraised separately using the JBI checklist. Both studies clearly defined the intervention and outcomes, used repeated and reliable outcome measurements, and applied appropriate statistical analyses. However, neither included a concurrent control group, and the comparability of the two non-randomized BERENICE cohorts was unclear. These limitations restricted causal and comparative inference; therefore, both studies were used only as contextual evidence. The complete JBI assessment is presented in Supplementary Table S3.

### 3.4. Narrative Synthesis

The treatment landscape of early HER2-positive breast cancer has evolved substantially over the past two decades. The included studies reflected this transition from anthracycline-containing chemotherapy toward increasingly individualized HER2-targeted approaches. The introduction of HER2-targeted therapies fundamentally changed clinical management by incorporating dual HER2 blockade, antibody–drug conjugates, and response-adapted treatment approaches. Across the included studies, these strategies were associated with improved oncologic outcomes while providing opportunities for treatment de-escalation in selected patient populations. NeoSphere demonstrated that adding pertuzumab to trastuzumab and docetaxel significantly improved pathological complete response rates without a meaningful increase in cardiac adverse events, whereas APHINITY reported improved invasive disease-free survival with dual HER2 blockade, particularly in patients with node-positive disease, while maintaining a favorable cardiac safety profile [13,19].

#### 3.4.1. Cardiac Safety of Anthracycline-Containing and Anthracycline-Free Regimens

Across the included studies, anthracycline-containing regimens were generally associated with higher rates of cardiac dysfunction than anthracycline-free strategies, although severe cardiac events remained uncommon with contemporary cardiac monitoring. The BCIRG-006 trial, one of the largest randomized trials included in this review, reported more frequent declines in LVEF and symptomatic heart failure among patients receiving anthracycline-containing therapy (AC → TH) than among those treated with the non-anthracycline TCH regimen [9]. Similarly, the primary and 3-year follow-up analyses of the TRAIN-2 trial demonstrated comparable oncologic outcomes regardless of chemotherapy backbone, while clinically relevant LVEF decline occurred significantly more frequently in the anthracycline-containing treatment group [10,11].

The TRYPHAENA trial further supported the cardiac safety of modern HER2-directed treatment strategies. Although decreases in LVEF were observed across all treatment groups, symptomatic left ventricular systolic dysfunction remained uncommon irrespective of chemotherapy backbone, including with dual HER2 blockade when accompanied by appropriate cardiac monitoring [12]. Likewise, BERENICE reported low frequencies of severe cardiac events despite the use of anthracycline-based neoadjuvant chemotherapy combined with trastuzumab and pertuzumab [14].

#### 3.4.2. Anthracycline-Sparing Strategies and Treatment De-Escalation

Several studies evaluated treatment de-escalation strategies designed to reduce treatment-related toxicity while maintaining oncologic efficacy. The APT trial reported excellent long-term oncologic outcomes together with a low incidence of cardiac events in patients with small, node-negative HER2-positive breast cancer treated with paclitaxel plus trastuzumab [17]. Similarly, the ATEMPT trial reported favorable efficacy and acceptable cardiac safety with adjuvant T-DM1 compared with paclitaxel plus trastuzumab [21].

The Short-HER trial compared 9 weeks versus 1 year of trastuzumab administered with chemotherapy. Although the shorter trastuzumab regimen did not demonstrate non-inferiority for disease-free survival, fewer cardiac events were reported than with the standard one-year regimen [18].

PHERGain demonstrated the feasibility of PET-guided response-adapted treatment, allowing selected patients to omit conventional chemotherapy while maintaining favorable three-year invasive disease-free survival [16].

#### 3.4.3. Novel HER2-Targeted Therapies and Individualized Treatment

Several studies evaluated antibody–drug conjugates as alternatives to conventional HER2-directed treatment strategies. In KATHERINE, adjuvant T-DM1 significantly improved invasive disease-free survival among patients with residual disease after neoadjuvant therapy while maintaining a low incidence of cardiac adverse events [20]. Conversely, KRISTINE demonstrated lower overall toxicity with T-DM1 plus pertuzumab than with conventional TCHP, although pathological complete response rates were lower [15]. These findings further support treatment individualization while maintaining favorable cardiac safety.

**Figure 3:**
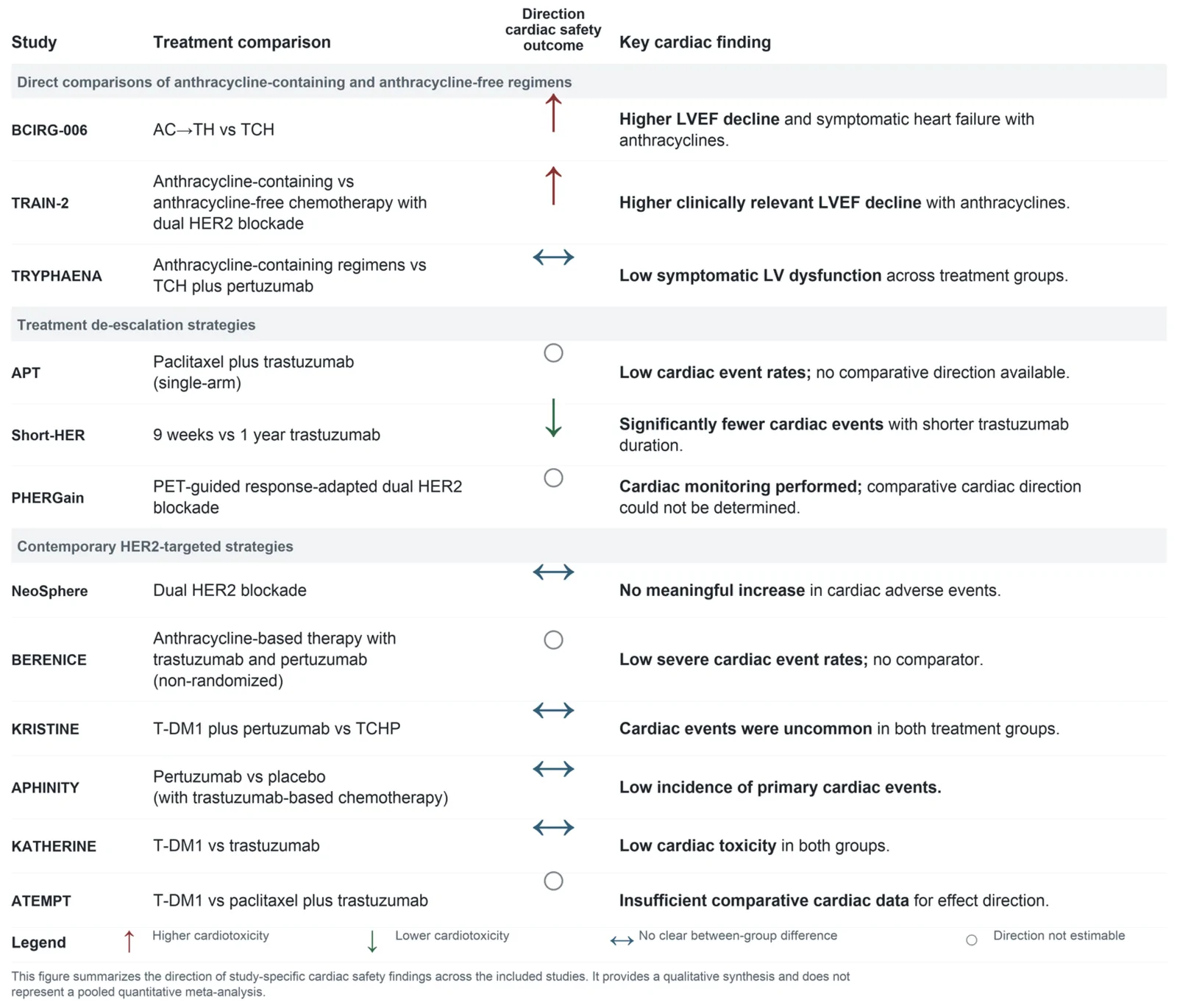
Effect direction plot of cardiac safety findings across the included studies. Arrows indicate the direction of study-specific cardiac safety outcomes relative to the study-specific comparator (↑ higher cardiotoxicity; ↓ lower cardiotoxicity; ↔ no clear between-group difference; ○ direction not estimable). The figure provides a qualitative summary of the included evidence and does not represent a pooled quantitative meta-analysis. The symbols represent qualitative interpretation of study-specific findings rather than effect size, statistical significance, or quantitative treatment estimates.

The overall direction of cardiac safety findings across the included studies is summarized in Figure 3, providing a visual complement to the narrative synthesis.

### 3.5. Certainty of Evidence

The certainty of evidence for the direct comparative cardiac outcomes ranged from low to moderate. For LVEF decline, the certainty of evidence was rated as low, with downgrading for inconsistency and imprecision. For CHF/symptomatic LVSD, the certainty of evidence was rated as moderate, with downgrading for imprecision. Risk of bias and indirectness were not considered serious enough to warrant downgrading for either outcome. Publication bias could not be formally assessed because only three direct comparative trials contributed to the evidence base. Details of the GRADE assessment are summarized in Table 3.

## 4. Discussion

The present systematic review evaluated the evolving role of anthracyclines in patients with early HER2-positive breast cancer receiving HER2-targeted therapy. Evidence from the three direct comparative trials generally indicated greater cardiac toxicity with anthracycline-containing regimens, although severe cardiac events were uncommon and the magnitude of risk varied across studies. The broader contextual evidence demonstrated favorable oncologic outcomes and generally low rates of cardiac events with several contemporary anthracycline-sparing and treatment de-escalation strategies in selected patient populations. Because most contextual studies did not directly isolate the effect of anthracycline exposure, these findings should be interpreted as supportive rather than comparative evidence.

These findings have important clinical implications because contemporary treatment decisions require balancing oncologic efficacy against potential long-term cardiovascular toxicity. However, the observed cardiac event rates cannot be compared indiscriminately across all included studies because the trials differed in patient risk, treatment setting, therapeutic regimens, cardiac monitoring, and follow-up duration. The direct comparative trials also applied divergent definitions of cardiotoxicity. BCIRG-006 defined LVEF decline as a sustained relative reduction of >10% from baseline [9], whereas TRAIN-2 and TRYPHAENA defined LVEF decline as an absolute decrease of ≥10 percentage points from baseline to <50% [10,12]. BCIRG-006 additionally reported NYHA class III–IV congestive heart failure, with suspected cardiac events reviewed by an independent cardiac review panel [9], whereas TRAIN-2 and TRYPHAENA reported symptomatic left ventricular systolic dysfunction as a separate cardiac outcome [10,12]. These differences in cardiac endpoint definitions, together with variation in cardiac monitoring schedules and the small direct comparative evidence base, reduced comparability across trials and precluded a clinically meaningful quantitative synthesis. The direct comparative evidence informs the cardiac trade-off associated with anthracycline exposure, whereas the contextual evidence illustrates the expanding range of treatment strategies available for selected patient populations. Established biological mechanisms of anthracycline-associated cardiotoxicity also support the observed differences in cardiac safety.

The biological plausibility of these findings is supported by well-established mechanisms of cardiotoxicity. Anthracycline-induced cardiac injury is cumulative, dose-dependent, and may result in irreversible myocardial damage through oxidative stress, mitochondrial dysfunction, and topoisomerase-IIβ-mediated pathways [22]. In contrast, trastuzumab interferes with HER2 signaling involved in cardiomyocyte survival and repair, thereby reducing the myocardium’s ability to recover from prior injury [23]. These complementary mechanisms provide a biological rationale for the higher rates of cardiac dysfunction observed when anthracyclines and HER2-targeted therapies are used sequentially or in combination. Such interactions are well recognized in the cardio-oncology literature and support the concept of cumulative myocardial injury associated with anthracycline exposure [24,25].

Taken together, BCIRG-006 and TRAIN-2 suggest that the incremental oncologic benefit of anthracyclines may be limited for many patients receiving effective HER2-targeted therapy, whereas the increased risk of cardiotoxicity remains clinically relevant. These findings are consistent with current international guidelines supporting individualized, risk-adapted treatment selection and consideration of anthracycline-sparing strategies in appropriately selected patients [26,27,28].

Our findings are consistent with the previous systematic review and meta-analysis by Zhu et al., which found no significant improvement in pathological complete response or breast-conserving surgery with anthracycline-containing neoadjuvant regimens but reported a higher incidence of LVEF decline [6]. However, the two reviews address related but distinct clinical questions. Zhu et al. focused on direct comparisons in the neoadjuvant setting and included both randomized and retrospective studies, whereas the present review considered prospective evidence across neoadjuvant and adjuvant settings. Furthermore, our synthesis prioritized cardiac safety and incorporated contemporary anthracycline-sparing, antibody–drug conjugate, dual HER2-blockade, and response-adapted treatment strategies. Because only three included trials directly compared anthracycline-containing and anthracycline-free regimens, these comparative findings were interpreted separately from the broader contextual evidence and were not quantitatively pooled. Thus, the present review complements the previous meta-analysis by placing its findings within the evolving clinical landscape of HER2-targeted treatment and de-escalation rather than attempting to replicate its quantitative conclusions.

Real-world observational studies have also reported increased risks of cardiomyopathy and heart failure among patients treated with anthracycline- and trastuzumab-based therapies. Large population-based cohort studies demonstrated higher rates of congestive heart failure in patients receiving trastuzumab, particularly when administered after anthracycline chemotherapy [29]. More recent real-world studies similarly suggest that combined exposure to anthracyclines and HER2-targeted therapy substantially increases the risk of cardiotoxicity compared with non-anthracycline regimens [30,31].

The evolution of HER2-targeted therapy has fundamentally altered the risk–benefit balance of anthracycline use. Historically, anthracyclines were considered essential components of systemic treatment because of their proven antitumor efficacy. However, the introduction of dual HER2 blockade, antibody–drug conjugates, and response-adapted treatment strategies has substantially improved outcomes while reducing dependence on anthracycline-containing chemotherapy. Trials such as APT demonstrated excellent long-term outcomes with paclitaxel and trastuzumab in patients with small node-negative tumors [17], whereas PHERGain showed that selected patients could safely omit chemotherapy within a response-adapted treatment strategy while maintaining favorable invasive disease-free survival [16]. In higher-risk populations, KATHERINE established adjuvant T-DM1 as an effective escalation strategy for patients with residual disease following neoadjuvant therapy [20]. These studies support treatment strategies that may reduce unnecessary exposure to anthracyclines while maintaining favorable oncologic outcomes in appropriately selected patients. However, APT and PHERGain were single-arm or response-adapted studies without a concurrent anthracycline-containing comparator; therefore, they support the feasibility of reduced-intensity strategies in selected patient populations but cannot establish oncologic equivalence or non-inferiority to anthracycline-containing regimens. Accordingly, statements regarding the comparative oncologic effect of anthracycline exposure in this review are restricted to the three direct comparative trials (BCIRG-006, TRAIN-2, and TRYPHAENA).

An important consideration is that several contemporary trials supporting anthracycline-sparing approaches, including TRAIN-2 and TRYPHAENA, were conducted in the neoadjuvant setting and enrolled selected patient populations. Therefore, extrapolation of these findings to all patients with HER2-positive early breast cancer should be undertaken with appropriate caution.

These findings do not support the universal abandonment of anthracyclines. Anthracycline-containing regimens may remain appropriate for selected patients in whom the anticipated oncologic benefit is considered to outweigh the potential cardiovascular risk. However, the available direct comparative trials do not establish precisely which patient subgroups derive additional benefit from anthracycline exposure. Treatment selection should therefore incorporate disease stage, nodal status, tumor biology, previous treatment response, baseline cardiovascular risk, and patient preferences. Contemporary breast cancer and cardio-oncology guidelines similarly emphasize individualized treatment selection, baseline cardiovascular risk assessment, structured surveillance, and early detection of cancer therapy-related cardiac dysfunction [24,25,32,33].

From a clinical perspective, these findings reinforce the importance of shared decision-making. Many patients with early HER2-positive breast cancer are expected to achieve long-term survival; consequently, treatment-related cardiovascular morbidity may have important implications even when severe cardiac events are uncommon. Patients should be informed about the expected oncologic benefits, potential cardiac risks, available anthracycline-sparing alternatives, and uncertainties in the comparative evidence. Integrating cardiovascular risk assessment and patient preferences into treatment planning may help optimize both oncologic and long-term health outcomes.

This systematic review has several strengths. It provides a comprehensive synthesis of available evidence regarding the role of anthracyclines in early HER2-positive breast cancer while integrating data from randomized trials evaluating both conventional anthracycline-containing regimens and emerging anthracycline-sparing strategies. By synthesizing evidence narratively, this review places cardiotoxicity findings within the broader context of evolving HER2-targeted therapies, treatment de-escalation, and clinical decision-making. The findings are consistent with the growing emphasis on risk-adapted treatment strategies reflected in contemporary breast cancer and cardio-oncology guidelines [26,27,28].

### 4.1. Limitations

Several limitations should be acknowledged. First, only three trials directly compared anthracycline-containing and anthracycline-free regimens, limiting the strength and precision of comparative conclusions. The remaining nine studies provided contextual evidence on contemporary HER2-targeted and treatment de-escalation strategies but did not directly isolate the effect of anthracycline exposure. Second, the included studies were heterogeneous with respect to clinical setting, patient population, treatment regimen, duration of follow-up, definitions of cardiotoxicity, cardiac imaging modalities, and monitoring schedules. This heterogeneity, together with the limited direct comparative evidence base, precluded a clinically meaningful quantitative synthesis. Third, several trials enrolled highly selected patient populations, which may limit generalizability to routine clinical practice and to patients with substantial baseline cardiovascular risk or comorbidity. Fourth, the change from the initially planned meta-analysis to a structured narrative synthesis represented a deviation from the original analytical plan, although this change has been reported transparently. Finally, the review included only English-language publications and did not generate a pooled effect estimate or permit formal assessment of publication bias. In addition, the literature search was limited to PubMed, the Web of Science, and CENTRAL and did not include Embase or systematic searches of trial registries. Although we manually screened reference lists of relevant articles, we may have missed potentially eligible unpublished or non-indexed studies.

### 4.2. Implications for Future Research

Future research incorporating high-quality observational data may help better characterize cardiotoxicity risk across broader, more representative patient populations. Although the present analysis focuses on clinical trial evidence, emerging research in cardio-oncology and precision medicine highlights advanced risk-prediction approaches, including imaging biomarkers and machine-learning-based models. Studies have shown that myocardial strain imaging and artificial intelligence-based models may improve early detection and prediction of cancer therapy-related cardiotoxicity [34,35,36]. While these approaches remain outside the scope of the current analysis, they represent a direction for future research to identify patients at the highest risk of cardiac complications during HER2-targeted therapy.

Future research should also prioritize the development of standardized definitions of cardiotoxicity and harmonized cardiac monitoring protocols across clinical trials to improve comparability of results. In parallel, ongoing clinical programs such as COMPASS-HER2 are evaluating response-adapted and risk-adapted treatment strategies across different HER2-positive breast cancer populations. Results from these studies may further refine patient selection and help identify patients who can safely receive less intensive systemic therapy [37]. Future studies should evaluate whether clinical, cardiovascular, genomic, and molecular risk factors can identify patients most likely to derive meaningful oncologic benefit from anthracycline-containing regimens while minimizing unnecessary cardiotoxicity. In addition, individual-patient data meta-analyses are needed to enable more precise risk stratification and to evaluate the impact of key modifiers, such as age, baseline cardiovascular risk, cumulative anthracycline dose, and treatment sequencing.

None of the included trials was specifically designed to evaluate a protocolized primary cardioprotective intervention. Although several trial protocols incorporated cardiac surveillance based primarily on serial LVEF assessment, baseline biomarker-guided surveillance using cardiac troponins or natriuretic peptides was not systematically incorporated across the included evidence base. This review could not evaluate the comparative effectiveness of primary cardioprotective strategies and biomarker-guided surveillance.

Integrating cardiac biomarkers, such as troponins and natriuretic peptides, with advanced imaging techniques may improve early detection of subclinical cardiac dysfunction. Prospective studies should also further evaluate the potential role of cardioprotective strategies, including angiotensin-converting enzyme inhibitors, SGLT-2 inhibitors, and beta-blockers.

Long-term survivorship outcomes deserve greater attention, as late heart failure may develop several years after completion of cancer therapy and may not be fully captured within the follow-up periods of randomized clinical trials. Integration of cardio-oncology principles into breast cancer management may ultimately help optimize both therapeutic efficacy and long-term cardiovascular health. Such approaches may further support more evidence-based, precise treatment decisions in HER2-positive breast cancer.

## 5. Conclusions

This systematic review highlights the changing role of anthracyclines in early HER2-positive breast cancer in the era of contemporary HER2-targeted therapy. Evidence from three direct comparative trials generally suggested greater cardiac toxicity with anthracycline-containing regimens, although differences in study design, treatment setting, and cardiotoxicity definitions limited quantitative interpretation. The broader contextual evidence supports the feasibility of several anthracycline-sparing and treatment de-escalation strategies in selected patient populations but does not establish their universal equivalence to anthracycline-containing treatment. The available evidence therefore supports selective rather than routine use of anthracyclines, guided by the anticipated oncologic benefit, baseline cardiovascular risk, tumor characteristics, treatment setting, and patient preferences. Further direct comparative studies with standardized cardiac outcomes and long-term follow-up are required to identify patients most likely to benefit from anthracycline-containing therapy.

## Figures and Tables

**Table 1:** Characteristics of studies included in the systematic review.

Study	Country/Setting	Study Design	Population	Sample Size	Treatment Groups	Cardiac Outcome Assessed
BCIRG-006 [9]	Multinational	Phase III randomized controlled trial	HER2-positive early breast cancer	3222	Anthracycline-based regimen (AC → TH) vs. non-anthracycline regimen (TCH)	Symptomatic heart failure; LVEF decline
TRAIN-2 [10,11]	Netherlands (37 hospitals)	Phase III randomized open-label trial	Stage II–III HER2-positive breast cancer	438	Anthracycline-containing regimen vs. anthracycline-free regimen with dual HER2 blockade	LVEF decline; symptomatic LV dysfunction
TRYPHAENA [12]	Multinational	Phase II randomized trial	HER2-positive early breast cancer receiving neoadjuvant therapy	225	Anthracycline-containing vs. non-anthracycline trastuzumab-based regimens	LVEF decline; symptomatic heart failure
NeoSphere [13]	Multinational	Phase II randomized trial	HER2-positive early breast cancer (neoadjuvant)	417	Trastuzumab + docetaxel vs. pertuzumab + trastuzumab + docetaxel vs. pertuzumab + trastuzumab vs. pertuzumab + docetaxel	Cardiac adverse events; LVEF decline
BERENICE [14]	Multinational multicenter study	Phase II open-label, non-randomized multicenter study	HER2-positive early breast cancer receiving neoadjuvant therapy	401	Cohort A: dose-dense doxorubicin + cyclophosphamide → paclitaxel + trastuzumab + pertuzumab; Cohort B: FEC → docetaxel + trastuzumab + pertuzumab	NYHA class III/IV heart failure; LVEF decline
KRISTINE [15]	Multinational, multicenter study	Phase III randomized trial	Stage II–III HER2-positive breast cancer	444	T-DM1 plus pertuzumab vs. docetaxel, carboplatin, trastuzumab, and pertuzumab (TCHP)	LVEF decrease; cardiac failure
PHERGain [16]	Multinational	Phase II randomized open-label trial	Stage I–IIIA HER2-positive early breast cancer	356	Conventional TCHP vs. PET-guided, response-adapted dual HER2 blockade with selective chemotherapy use	Cardiac adverse events; LVEF monitoring
APT [17]	Multicenter	Phase II single-arm trial	Node-negative HER2-positive early breast cancer	406	Paclitaxel plus trastuzumab; prospective single-arm regimen	Symptomatic heart failure; LVEF decline
Short-HER [18]	Multinational	Phase III randomized non-inferiority trial	HER2-positive early breast cancer	1254	9 weeks versus 1 year of adjuvant trastuzumab administered with chemotherapy	Composite cardiac adverse events, including LVEF decline and symptomatic congestive heart failure
APHINITY [19]	Multinational	Phase III randomized double-blind trial	HER2-positive early breast cancer (adjuvant)	4805	Chemotherapy + trastuzumab + pertuzumab vs. chemotherapy + trastuzumab	Primary cardiac events; LVEF decline
KATHERINE [20]	Multinational	Phase III randomized trial	HER2-positive early breast cancer with residual disease after neoadjuvant therapy	1486	T-DM1 vs. trastuzumab	Symptomatic heart failure; LVEF decline
ATEMPT [21]	Multicenter	Phase II randomized trial	Stage I HER2-positive breast cancer	512	T-DM1 for 1 year vs. paclitaxel + trastuzumab (TH)	Cardiac adverse events; LVEF decline

**Note**: The table summarizes study design, patient population, sample size, treatment regimens, and reported cardiac outcomes. BCIRG-006 was a three-arm trial (AC → T, AC → TH, and TCH); the reported sample size (N = 3222) represents the full trial population across all three treatment arms. Only the AC → TH and TCH arms, which differed with respect to anthracycline exposure, were included in the direct comparative synthesis in this review (see Table 2).

**Table 2:** Definitions of cardiotoxicity and cardiac monitoring characteristics across the direct comparative trials.

Study	Treatment Arm	Reported Cardiac Events (n/N)	Definition of Cardiotoxicity	LVEF Criterion	Cardiac Imaging Modality	Cardiac Monitoring Schedule	Follow-Up
BCIRG-006	AC → TH	LVEF decline: 194/1042; CHF: 21/1074	Sustained subclinical loss of mean LVEF defined as a relative reduction >10% from baseline at the last follow-up evaluation; CHF adjudicated by an independent cardiac review panel	>10% relative reduction from baseline	Serial LVEF assessment (imaging modality not consistently specified)	LVEF assessed seven times during the study	Median 65 months
BCIRG-006	TCH	LVEF decline: 97/1031; CHF: 4/1075	Same as above	Same	Same	Same	Median 65 months
TRAIN-2	Anthracycline-containing regimen	LVEF decline: 17/220; symptomatic LVSD 2/220	Decline in LVEF ≥10% from baseline to <50% at any time	≥10% decrease to <50%	Echocardiography	Every 3 months during ERBB2 blockade	Median 48.8 months
TRAIN-2	Non-anthracycline regimen	LVEF decline: 7/218; symptomatic LVSD 0/218	Same	Same	Echocardiography	Every 3 months during ERBB2 blockade	Median 48.8 months
TRYPHAENA	Arm A (FEC + H + P → T + H + P)	LVEF decline: 4/72	LVEF decline ≥10 percentage points from baseline to <50% during neoadjuvant therapy	≥10 percentage points to <50%	Echocardiography or MUGA (same modality used throughout for each patient)	Baseline; cycles 2, 4, and 6; before cycle 7; during cycles 10, 12, 15, and 18 (Arm B only); final visit/withdrawal; every 6 months for 2 years, then annually for 2 years	Median overall study duration 20–21 months
TRYPHAENA	Arm B (FEC → T + H + P)	LVEF decline: 4/75; symptomatic LVSD: 2/75	Symptomatic LVSD and/or LVEF decline ≥10 percentage points from baseline to <50%	≥10 percentage points to <50%	Echocardiography or MUGA	Same as above	Median overall study duration 20–21 months
TRYPHAENA	Arm C (TCH + P)	LVEF decline: 3/76	LVEF decline ≥10 percentage points from baseline to <50% during neoadjuvant therapy	≥10 percentage points to <50%	Echocardiography or MUGA	Same as above	Median overall study duration 20–21 months

**Abbreviations**: AC, doxorubicin plus cyclophosphamide; CHF, congestive heart failure; FEC, fluorouracil, epirubicin, and cyclophosphamide; H, trastuzumab; LVEF, left ventricular ejection fraction; LVSD, left ventricular systolic dysfunction; MUGA, multigated acquisition scan; P, pertuzumab; T, docetaxel; TCH, docetaxel, carboplatin, and trastuzumab. **Note**: For BCIRG-006, the denominators for congestive heart failure correspond to the full trial-group populations (AC→TH: *n* = 1074; TCH: *n* = 1075), whereas the denominators for LVEF decline reflect patients with evaluable LVEF assessments (AC→TH: *n* = 1042; TCH: *n* = 1031), as reported in the original trial publication [9].

**Table 3:** GRADE summary of the certainty of evidence for the direct comparative cardiac outcomes (BCIRG-006, TRAIN-2, TRYPHAENA).

Outcome	Studies	Risk of Bias	Inconsistency	Indirectness	Imprecision	Certainty
LVEF decline	3 RCTs	Not serious *	Serious **^1^**	Not serious	Serious **^2^**	⊕⊕○○ Low
CHF/symptomatic LVSD	3 RCTs	Not serious *	Not serious	Not serious	Serious **^3^**	⊕⊕⊕○ Moderate

* Although TRAIN-2 and TRYPHAENA were open-label trials, the identified risk-of-bias concerns were not considered sufficient to warrant downgrading the certainty of evidence for the cardiac outcomes. **^1^** Downgraded one level for inconsistency because of clinical and methodological heterogeneity across trials, including differences in treatment regimens, cardiac monitoring, follow-up, and definitions of LVEF decline. **^2^** Downgraded one level for imprecision because the comparative evidence was limited to three trials, with relatively few LVEF-decline events in TRAIN-2 and TRYPHAENA and no pooled effect estimate. **^3^** Downgraded one level for imprecision because clinically overt CHF/symptomatic LVSD events were uncommon across the direct comparative trials, resulting in uncertainty around the magnitude of the comparative effect. GRADE certainty ratings: ⊕⊕○○ = low certainty; ⊕⊕⊕○ = moderate certainty.

## Data Availability

All data analyzed in this systematic review were obtained from publicly available published studies. The complete search strategies, study-level data, and risk-of-bias assessments are provided in the article and its Electronic Supplementary Material.

## Supplementary Materials

The following supporting information can be downloaded at https://www.mdpi.com/article/10.3390/jcm15187303/s1, Supplementary Table S1. Detailed search strategy. Supplementary Table S2. Risk-of-bias assessment of randomized controlled trials using the Cochrane Risk of Bias 2 tool. Supplementary Table S3. JBI critical appraisal of prospective non-randomized intervention studies.
