# Supplementary material for: Reassessing the Role of Anthracyclines in Early HER2-Positive Breast Cancer in the Era of HER2-Targeted Therapy and Treatment De-Escalation: A Systematic Review and Narrative Synthesis
Source: J Clin Med. 2026 Sep 20;15(18):7303. doi: 10.3390/jcm15187303 (PMC13607463; doi:10.3390/jcm15187303)
Supplement: Supplementary file 1 [file jcm-15-07303-s001.zip › jcm-4548887-supplementary.pdf]

Supplementary Table S1. Detailed search strategy

**PubMed (Title/Abstract):**	(breast cancer OR breast neoplasm*) AND (HER2 OR erbB2) AND trastuzumab AND anthracycline* AND (cardiotoxicity OR "heart failure" OR "cardiac dysfunction" OR LVEF OR "left ventricular ejection fraction")
**Web of Science (Topic search):**	TS = ((breast cancer OR breast neoplasm*) AND (HER2 OR erbB2) AND trastuzumab AND anthracycline* AND (cardiotoxicity OR "heart failure" OR "cardiac dysfunction" OR LVEF OR "left ventricular ejection fraction"))
**Cochrane CENTRAL (Title, Abstract, Keyword):**	(breast cancer OR breast neoplasm*) AND (HER2 OR erbB2) AND trastuzumab AND anthracycline* AND (cardiotoxicity OR "heart failure" OR "cardiac dysfunction" OR LVEF OR "left ventricular ejection fraction")

Supplementary Table S2. Risk of bias assessment of randomized controlled trials using the Cochrane Risk of Bias 2 tool

Study	Randomization process	Deviations from intended interventions	Missing outcome data	Measurement of the outcome	Selection of reported result	Overall risk of bias
BCIRG-006	Low	Low	Low	Low	Low	Low
TRAIN-2	Low	Some concerns	Low	Low	Low	Some concerns
TRYPHAENA	Low	Some concerns	Low	Low	Low	Some concerns
NeoSphere	Low	Some concerns	Low	Low	Low	Some concerns
KRISTINE	Low	Some concerns	Low	Low	Low	Some concerns
PHERGain	Low	Some concerns	Low	Low	Low	Some concerns
Short-HER	Low	Low	Low	Low	Low	Low

APHINITY	Low	Low	Low	Low	Low	Low
KATHERINE	Low	Low	Low	Low	Low	Low
ATEMPT	Low	Some concerns	Low	Low	Low	Some concerns

Supplementary Table S3. JBI critical appraisal of prospective non-randomized intervention studies

JBI appraisal item	BERENICE	APT
1. Is it clear in the study what is the "cause" and what is the "effect"?	Yes	Yes
2. Were the participants included in any comparisons similar?	Unclear	N/A
3. Were the participants included in any comparisons receiving similar treatment/care, other than the exposure or intervention of interest?	Yes	N/A
4. Was there a control group?	No	No
5. Were there multiple measurements of the outcome both before and after the intervention?	Yes	Yes
6. Was follow-up complete and, if not, were differences between groups adequately described and analyzed?	Yes	Yes
7. Were the outcomes of participants measured in the same way?	Yes	Yes
8. Were outcomes measured reliably?	Yes	Yes
9. Was appropriate statistical analysis used?	Yes	Yes

Abbreviations: JBI, Joanna Briggs Institute; N/A, not applicable. BERENICE was a prospective, non-randomized, multicenter phase II study with two treatment cohorts, whereas APT was a prospective, single-arm phase II trial. The absence of a concurrent control group limited causal and comparative inference in both studies. Consequently, these studies were used as contextual evidence and not as direct estimates of the comparative effect of anthracycline exposure.
